# Printable and Stretchable Giant Magnetoresistive Sensors for Highly Compliant and Skin‐Conformal Electronics

**DOI:** 10.1002/adma.202005521

**Published:** 2021-02-02

**Authors:** Minjeong Ha, Gilbert Santiago Cañón Bermúdez, Tobias Kosub, Ingolf Mönch, Yevhen Zabila, Eduardo Sergio Oliveros Mata, Rico Illing, Yakun Wang, Jürgen Fassbender, Denys Makarov

**Affiliations:** ^1^ Institute of Ion Beam Physics and Materials Research Helmholtz‐Zentrum Dresden‐Rossendorf e.V. Bautzner Landstrasse 400 Dresden 01328 Germany; ^2^ The Henryk Niewodniczanski Institute of Nuclear Physics Polish Academy of Sciences Krakow 31‐342 Poland

**Keywords:** magnetoresistive materials, printable materials, sensors, skin‐conformal materials, stretchable materials

## Abstract

Highly compliant electronics, naturally conforming to human skin, represent a paradigm shift in the interplay with the surroundings. Solution‐processable printing technologies are yet to be developed to comply with requirements to mechanical conformability of on‐skin appliances. Here, it is demonstrated that high‐performance spintronic elements can be printed on ultrathin 3 µm thick polymeric foils enabling the mechanically imperceptible printed magnetoelectronics, which can adapt to the periodic buckling surface to be biaxially stretched over 100%. They constitute the first example of printed and stretchable giant magnetoresistive sensors, revealing 2 orders of magnitude improvements in mechanical stability and sensitivity at small magnetic fields, compared to the state‐of‐the‐art printed magnetoelectronics. The key enabler of this performance enhancement is the use of elastomeric triblock copolymers as a binder for the magnetosensitive paste. Even when bent to a radius of 16 µm, the sensors printed on ultrathin foils remain intact and possess unmatched sensitivity for printed magnetoelectronics of 3 T^‐1^ in a low magnetic field of 0.88 mT. The compliant printed sensors can be used as components of on‐skin interactive electronics as it is demonstrated with a touchless control of virtual objects including zooming in and out of interactive maps and scrolling through electronic documents.

## Graphical abstract

Highly compliant and printable giant magnetoresistive sensors reveal skin‐conformability and excellent sensitivity to small magnetic fields because of highly sensitive giant magnetoresistive multilayers bound with sticky triblock copolymer, which is capable of on‐skin interactive electronics for a touchless control of virtual objects.

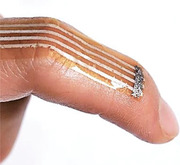

Rapid growth and high acceptance of flexible electronics are facilitated by a great demand for personalized services in the ubiquitous era such as personal health‐monitoring,^[^ ^1^, ^2^, ^3^ ^]^ electronic skins,^[^ ^4^, ^5^, ^6^ ^]^ soft robots,^[^ ^7^ ^]^ augmented reality,^[^ ^8^ ^]^ and internet of things (IoTs).^[^ ^9^ ^]^ Central to these customized appliances is the establishment of highly adaptable and skin‐conformal functional elements capable of recognizing environmental changes through all aspects of daily life or to track position, motion and gestures by responding to electrical,^[^ ^2^, ^10^ ^]^ magnetic,^[^ ^5^, ^6^, ^8^, ^11^ ^]^ optical,^[^ ^12^ ^]^ and thermal^[^ ^13^ ^]^ stimuli. Solution‐processable printing technologies are very attractive for the realization of human interactive and highly compliant devices as they are simple, cost‐efficient and adaptable to various materials at freely defined layouts for functional elements.^[^ ^14^, ^15^, ^16^, ^17^ ^]^ Recent reports on printed electronics reveal the possibility to prepare also stretchable printed sensors of mechanical properties (strain, force, pressure, and bending),^[^ ^18^, ^19^, ^20^, ^21^ ^]^ which are relevant for on‐skin applications in human‐interactive systems, artificial intelligence, advanced prosthetics, and humanoid robots.

To realize compliant electronics,^[^ ^22^ ^]^ the state of the art approaches rely on thin film deposition and lithographic processing of organic and inorganic materials directly on ultrathin polymeric foils.^[^ ^23^, ^24^, ^25^ ^]^ Exciting progress has been made in the direction of all‐printed stretchable electronics^[^ ^19^, ^26^ ^]^ and stretchable thin‐film magnetoelectronics.^[^ ^27^ ^]^ However, combining both printable and stretchable qualities for magnetoelectronic sensors has not yet been demonstrated. Among various mechanically imperceptible functional elements, compliant magnetic field sensors, with their action‐at‐a‐distance nature, enable touchless on‐skin interactivity relying on the surrounding magnetic fields, for applications ranging from human‐machine interaction to noninvasive medical diagnostics.^[^ ^5^, ^11^, ^28^ ^]^ In stark contrast to excellent mechanical and magnetoresistive performance of foil‐based magnetoelectronics, printed magnetosensitive devices^[^ ^29^, ^30^, ^31^, ^32^, ^33^ ^]^ are rather stiff, supporting bending down to radii of more than 1 cm only,^[^ ^30^ ^]^ and have so far been applied for the detection of high magnetic fields in the range of 100 mT. These high fields are unacceptable for on‐skin devices as the continuous exposure limit prescribed by the world health organization (WHO) is <40 mT.^[^ ^34^, ^35^ ^]^ Even for the best printed magnetic field sensors, which are based on the giant magnetoresistance (GMR) effect, the sensitivity in the relevant field range is rather poor. We note that the word “giant” is related to the physical mechanism behind the resistance change. To quantify the technological relevance of printed GMR sensors and to be able to compare different sensor technologies, it is insightful to define a figure of merit given by the ratio of the sensor maximum sensitivity (*S*_max_) to the field (*H*_S = max_) at which this sensitivity is reached. If this metric is considered, current printed GMR sensors feature a somewhat low figure of merit of about 7 T^–2^ only.^[^ ^30^ ^]^ The limitations in the case of printed magnetoelectronics are the consequence of the trade‐off between softness and sensing performance due to percolation networks of fillers in the printed magnetic paste.

Here, we realize printed GMR sensors for skin‐conformal interactive electronics. The sensors can be screen printed on ultrathin 3 µm thick polymeric foils and reveal a figure of merit of 3409 T^–2^ when operating in a low magnetic field range of about 0.88 mT (more than 2 orders of magnitude boost compared to the state of the art). Furthermore, printed sensors can be bent to 16 µm without sacrificing their magnetoresistive performance, resembling two orders of magnitude improvement in the mechanical stability compared to the previous reports. The highly compliant printed sensors follow well the radial buckling pattern on the stretchable substrates, resulting in stable magnetic‐field sensing capability under 100% of biaxial strain rate. The key enabler of the performance enhancement is the novel solution‐processable magnetic paste, which includes multilayered [Py/Cu]_30_ microflakes dispersed in triblock copolymer based poly(styrene‐butadiene‐styrene) (SBS) elastomer. The supramolecular structure of SBS possesses high dissipative capacity and cohesive tear strength, resulting in sufficiently strong interaction of interfacial carbon‐carbon covalent boding and great adhesion to the ultrathin polymeric foils.^[^ ^36^ ^]^ The use of the SBS elastomer as a binder assures unmatched mechanical stability even upon extreme bending due to excellent percolation between the [Py/Cu]_30_ microflakes. We are benefiting from the structure of the triblock copolymer, which is composed of a soft matrix and hard microdomains, providing high viscoelasticity for a tight adhesion to the curved surface. This viscoelastic adhesion contributes compact percolation contacts between the randomly distributed microflakes during volume shrinkage upon thermal annealing. The thermoplastic SBS allows spatial reconfiguration of randomly distributed GMR microflakes when SBS turns into a flow state with the increase of temperature, which contributes to enhancement of the overall GMR performance. To boost the magnetoresistive performance, for the first time in printed magnetoelectronics, we utilized GMR multilayered [Py/Cu]_30_ microflakes, which are coupled at the 2nd antiferromagnetic maximum. By using these weaker exchange coupled GMR multilayers, compared to the state‐of‐the‐art reports on printed magnetoelectronics, we successfully detect magnetic fields in the low‐field range of 0.88 mT. The printed [Py/Cu]_30_ microflakes based GMR sensors possess a high sensitivity *S*_max_ = 3 T^–1^ at 0.88 mT with a figure of merit, which is over 150 times higher than that of the best Co nanoparticle embedding gel based printable GMR sensors.^[^ ^29^ ^]^ Benefiting from their excellent mechanical compliancy, the screen printed GMR sensors on ultrathin foils can be conformally applied to human skin resulting in the very first printed yet mechanically imperceptible magnetoelectronics. We demonstrate the application of these printable on‐skin GMR sensors as remote control systems driven by small magnets, to scroll and zoom through documents on a PC. Furthermore, due to its printable, large‐area nature; this technology can be easily integrated in wearables and textiles for prospective consumer electronics and industrial applications.

The printed magnetoresistive sensor consists of [Py(1.5 nm)/Cu(2.3 nm)]_30_ microflakes mixed with viscoelastic SBS triblock copolymer. The sensor covers four printed electrodes to allow for a 4‐point resistance measurement as schematically illustrated in **Figure** **1**a. To prepare GMR microflakes, [Py/Cu]_30_ multilayers coupled at the 2nd antiferromagnetic maximum are grown on photoresist‐coated glass substrates by magnetron sputtering (Figure S1, Supporting Information). The as‐grown GMR stacks are transformed into microflakes by lift‐off and ultrasonication, followed by mixing the GMR powder with SBS binding elastomers to obtain a viscous magnetic paste. This GMR paste is screen printed directly on the ultrathin 3‐µm‐thick polymeric foils, such as polyimide and Mylar. The printed sensor is highly compliant and naturally conforms to skin and periodic curves on the joints of the wrist (Figure 1b,c). These pronouncedly curved regions of the skin typically attain bending radii in the order of tens of µm,^[^ ^26^ ^]^ which demands high mechanical stability on the sensor side.

**Figure 1:**
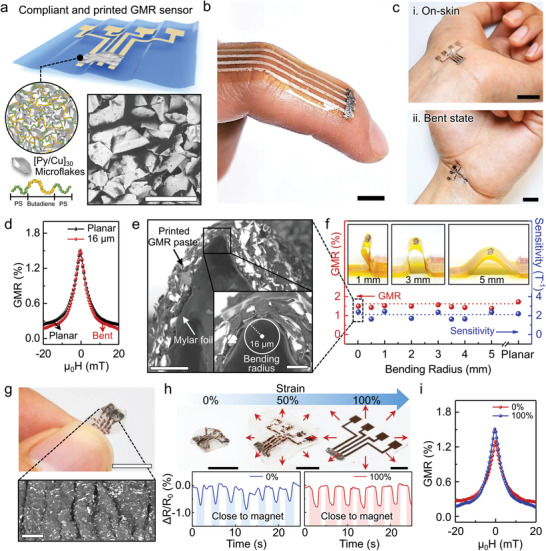
Highly compliant and printed GMR sensors. a) Schematic illustration of GMR sensors consisting of [Py/Cu]_30_ microflakes and triblock copolymer (SBS) printed on ultrathin foils. SEM image reveals dried GMR paste printed on a Mylar foil. Scale bar: 100 µm. b,c) Photographs of printed GMR sensors conformably applied on skin with curved body parts of a finger (b) and a stretched (c‐i) and bent (c‐ii) wrist. Scale bar: 1 cm. d) GMR performance of the printed sensors under planar and bent state (16 µm of bending radius). e) Cross‐sectional SEM images of bent GMR sensors printed on an ultrathin Mylar foil. At the apex the GMR sensor is bent to the radius of 16 µm (inset). Scale bars: 100 µm, 20 µm (inset). f) The magnitude of the GMR effect and the sensor sensitivity following the gradual decrease of the bending radius from 5 mm to 16 µm. g) Photograph of the printed GMR sensor laminated to a VHB tape, which was initially biaxially prestretched to 100%. The photo shows the sensor in its relaxed state. Scale bar: 1 cm. The bottom panel in (g) shows an SEM image of the buckled surface of the sensor. Scale bar: 300 µm. h) Photograph of a stretchable printed GMR sensor from 100% of stretching to 0% of relaxed state. Bottom panel in (h), shows the time evolution of the variation of the normalized sensor resistance for the sensor in the relaxed state (0%) and after the sensor is stretched to (100%). The sensor is exposed to a field of a permanent magnet, which is manually approached and retracted from the sensor, which leads to the resistance change. Scale bar: 5 mm. i) GMR performance of the printed sensor in the relaxed (0% stretching) and stretched state (100% stretching).

Due to the tackiness of soft polybutadiene chains in SBS triblock copolymer, GMR paste has an excellent adhesion to the extremely crumpled surface on the ultrathin Mylar foil (Figure 1a,e). Under a bending radius of 16 µm in the scanning electron microscopy (SEM) images (inset in Figure 1e), the printed GMR sensor showed unprecedented mechanical stability without delamination and disconnection of the GMR microflakes (Figure S1f–h, Supporting Information). During the drying process of printed GMR paste, the 3D distributed GMR microflakes in the fluidic pastes can be re‐organized and immobilized as randomly oriented 3D multi‐stacked layers due to the volumetric shrinkage of SBS.^[^ ^37^ ^]^ While the elastomers consisting of conventional composites mostly block to form good percolation networks between fillers because of the bulk occupation of viscous precursor liquid, the shrunken SBS induces the reorganization and firmly stacking of microflakes, to form a great interlayer electron transport and thus reduce the resistance level of printed GMR pastes. In addition, similar interaction of GMR microflakes with the polybutadiene and the polystyrene phases makes uniform distribution of microflakes in the polymer matrix regardless of composition,^[^ ^38^ ^]^ resulting in excellent percolation networks between the microflakes.

The shrunken SBS results in a relatively small thickness of films and strong links between the randomly oriented 3D‐stacked [Py/Cu]_30_ microflakes (Figure S1g, Supporting Information). We examined the morphology of GMR flakes after adding a 4 µL binder droplet over a Si substrate with sprinkled Permalloy/Cu flakes. When using SBS, the free surface energy assisted the formation of a compact core of flakes in the center of the droplet (Figure S2a,b, Supporting Information). Contrarily, the low wettability of polyepichlorohydrin (PCH), the binder that showed the best performance in the previous reports,^[^ ^30^ ^]^ hinders the formation of a compact network of flakes inside the droplet (Figure S2c,d, Supporting Information). Since the shrunken viscoelastic SBS stabilizes the percolation networks among the [Py/Cu]_30_ microflakes and gives high flexibility with excellent adhesion to polymeric foils, the printed GMR sensors display a similar GMR ratio (≈1.5%) under planar and bending states (bending radius: ≈16 µm), as shown in Figure 1d,e. The excellent mechanical stability of printed GMR sensors was caused by the sticking phase of microflakes bonded to the SBS matrix that enables deform together at the small bending strain. Under extreme bent state, energy dissipation occurs by slippage at the interface of microflakes and SBS matrix,^[^ ^39^ ^]^ which prevents the mechanical fracture and crack propagation of microflakes. We explored the effects of gradually bending the sensor foils to curvature radii between 5 mm and 16 µm and found that the GMR ratio (1.5 ± 0.1%) and magnetic field sensitivity (2.0 ± 0.3 T^–1^) are preserved even under these severe mechanical deformations (Figure 1e).

To test the stability of these compliant magnetic‐field sensing under biaxial stretching and compression, we attached them to VHB (very high bonding) tape, which we pre‐stretched and let relax. During the experiments, the VHB was initially stretched up to 100% of biaxial strain and fully released until the sensor displayed a complex buckled surface with an average curvature radius of 50 µm (Figure 1g). Cycling the sensors between 100% and 0% of strain did not affect the magnetic field detection capabilities of the printed GMR sensor. These conditions emulate the typically expected motion of the sensor if it were to be intimately attached to human skin (Figure 1c). Furthermore, the stretchable GMR sensor could easily detect the proximity of a ≈100 mT permanent magnet both in the strained and relaxed states (Figure 1h), while retaining its GMR performance (Figure 1i).

To reveal the mechanisms behind the suitability of using SBS as a binder to obtain stretchable magnetic sensors with stable magnetoelectric response, we analyzed the wettability and adhesion properties of the SBS on the Permalloy/Cu stacks (Figure S3, Supporting Information). We compared the properties of SBS with PCH. Using contact angle measurements over Permalloy/Cu stack as solid surface, we found a higher wettability of the SBS (12.5°) binder solution compared to PCH (41.2°). Furthermore, we address the cohesive properties of the binders (SBS vs PCH), we used PET foil as a flexible adherent probe and perform a peeling resistance test (Figure S4, Supporting Information). The high interfacial tension facilitates the mechanical contact between the surface of the GMR stack and a PET adherent probe. It was noted that SBS effectively absorbs the pulling forces due to the low cohesion provided by the physical crosslinks that are formed in the copolymer microstructure. PCH also damps the stress but fails abruptly at a lower peeling angle. This demonstrates that the viscoelastic properties of SBS make it more suitable for stretchable applications where the stresses during bending will be effectively absorbed by the copolymer reducing the chance of abrupt disconnection between flakes.

Primary consideration of on‐skin magnetic field sensors is a sensing capability at low magnetic fields (<40 mT, continuous exposure limit from WHO^[^ ^34^, ^35^ ^]^), which are acceptable for the human body on a daily basis. Exactly because of these concerns, the development of low‐field sensitive magnetic sensors is essential for practical use in wearable electronics. In this perspective, we characterize the sensor performance in terms of the technologically relevant parameter–the figure of merit (FoM). The sensitivity (*S*) of printed [Py/Cu]_30_ microflakes based GMR sensors, which is the first derivative of magnetoresistance (d*R*/d*H*) divided with saturated resistance (*R*_sat_), reaches a maximum value of ≈3 T^–1^ at 0.88 mT (**Table** **1** and Figure S5a, Supporting Information). The resulting FoM of 3409 T^–2^ is almost 150 times higher than for state‐of‐the‐art printed GMR sensors^[^ ^30^ ^]^ (**Figure** **2**b,e and Table 1), as required for the applications of wearable and on‐skin electronics. While current printed GMR sensors have insufficient FoM in the field range required for wearables, they still possess moderate sensitivity to relatively large magnetic fields (0.09 T^–1^ at 100 mT,^[^ ^32^ ^]^ 0.93 T^–1^ at 130 mT,^[^ ^30^ ^]^ 0.55 T^–1^ at 225 mT,^[^ ^31^ ^]^ 0.17 T^–1^ at 250 mT^[^ ^46^ ^]^ in Table 1), which are relevant for applications in industrial machines (Figure 2e). To address a broad range of magnetic field for numerous applications, this work aims to cover the gap of detectable field range between previous printed GMR sensors and the low field regime. Accordingly, we investigated GMR effect and magnetoresistive sensing performance of printed [Co/Cu]_50_ microflakes coupled at the 2nd antiferromagnetic maximum. This [Co/Cu]_50_ based GMR sensors represent GMR effect of 7% and comparable sensitivity (2 T^–1^) yet in the intermediate magnetic field (10 mT, Figure 2c,d and Figure S5b, Supporting Information), which gives a promise to apply them for consumer electronics (Figure 2e). Therefore, our printed GMR sensors based on efficient magnetic pastes will cover such broad application fields to overcome the limited usability of existing GMR sensors.

**Table 1:** Comparison of the mechanical and magnetoresistive performance of printed sensors. “TMR” stands for tunneling magnetoresistive effect

Type	Materials	Field of *S*max	Max magneto‐resistance	Sensitivity [*S*]	Figure of merits	Flexibility	Ref.
TMR	Self‐assembled Co nanocrystal	54 mT	8% (5 K)	0.17% mT^−1^	–	–	^[^ ^40^ ^]^
TMR	Self‐assembled Fe nanoparticles	≈250 mT	0.3% (RT)	0.023 T^–1^	0.09 T^–2^	–	^[^ ^41^ ^]^
TMR	Self‐assembled Fe_3_O_4_ nanoparticles	580 mT	7.3% (RT)	–	–	–	^[^ ^42^ ^]^
TMR	P3HT/Co nanoparticle composites	≈70 mT	2.9% (10 K)	0.004% mT^−1^	–	–	^[^ ^43^ ^]^
TMR	CoFe nanoparticles	4750 mT	3.1% (10 K)	0.0625 T^–1^	0.013 T^–2^	–	^[^ ^44^ ^]^
Magneto‐piezoresistance	Ag–PDMS/Fe−Ni alloy powder composites	65.4 mT	–	206% mT^–1^	–	Bendable (bending radius: 5 mm)	^[^ ^45^ ^]^
GMR	Co nanoparticles–gel composites	44 mT	260% (RT)	1.0 T^–1^	22.73 T^–2^	–	^[^ ^29^ ^]^
GMR	Co/Cu‐polypyrrole composites	≈100 mT	–	0.09 T^–1^	≈0.9 T^–2^	Flexible	^[^ ^32^ ^]^
GMR	FeCoNi/Cu nanowires	≈225 mT	14% (RT)	0.55 T^–1^	2.43 T^–2^	–	^[^ ^31^ ^]^
GMR	Printed Co/Cu flakes	≈250 mT	8% (RT)	0.17 T^–1^	0.68 T^–2^	Bendable	^[^ ^46^ ^]^
GMR	Printed Co/Cu flakes	130 mT	37% (RT)	0.93 T^–1^	7.15 T^–2^	Bendable (Bending radius: 12 mm)	^[^ ^30^ ^]^
GMR	Printed Py/Cu flakes	0.88 mT	1.95% (RT)	3.0 T^–1^, 0.43% mT^–1^	3409 T^–2^	Bendable (bending radius: 16 µm), Stretchable (100%)	This work

**Figure 2:**
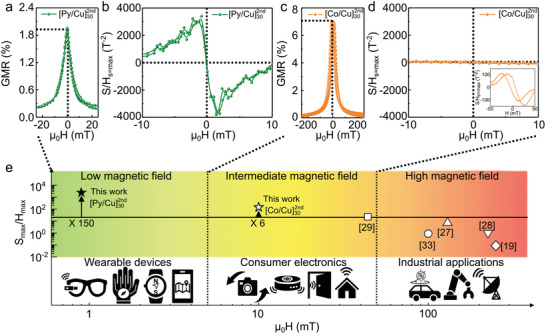
Figure of merits of printed GMR sensors. a–d) Sensing capability of printed GMR sensors based on Py/Cu (a,b) and Co/Cu (c,d) multilayer stacks in terms of GMR magnitude (a,c) and figure of merit (b,d). The inset in (d) shows the variations of the sensor sensitivity in the intermediate magnetic field range. e) Comparison of the figure of merits between the literature data (indicated references) and this work. The operation range of the sensors is correlated with application fields.

In comparison with thin film based GMR sensors, the randomly oriented [Py/Cu]_30_ microflakes in the printed GMR sensors enable omni‐directional magnetic field sensing depending on the in‐plane and out‐of‐plane field direction (**Figure** **3**a,b). Measuring the angular dependence of the printed GMR sensors from 0° to 360° reveals a constant, GMR ratio (2.09 ± 0.02%) over the entire range of angles screened (Figure 3c). Although printed GMR sensors mostly provide similar GMR ratio regardless of magnetic field direction, the highly aligned 2D‐stacked microflakes broaden GMR curves with degrading sensitivity at the out‐of‐plane field.^[^ ^46^ ^]^ In our approach, the tight bonding between GMR microflakes and SBS matrix favors the multi‐axial alignment of flakes over the typically encountered 2D stacking. As a result, this more random orientation of microflakes increases isotropic sensing without degrading sensitivity under in‐plane and out‐of‐plane field (Figure 3d). This omni‐directional sensing capability provides great benefits for freely locating GMR sensors on skin without special pre‐positioning of magnets, as skin‐mounted GMR sensors can adopt varying orientations.

**Figure 3:**
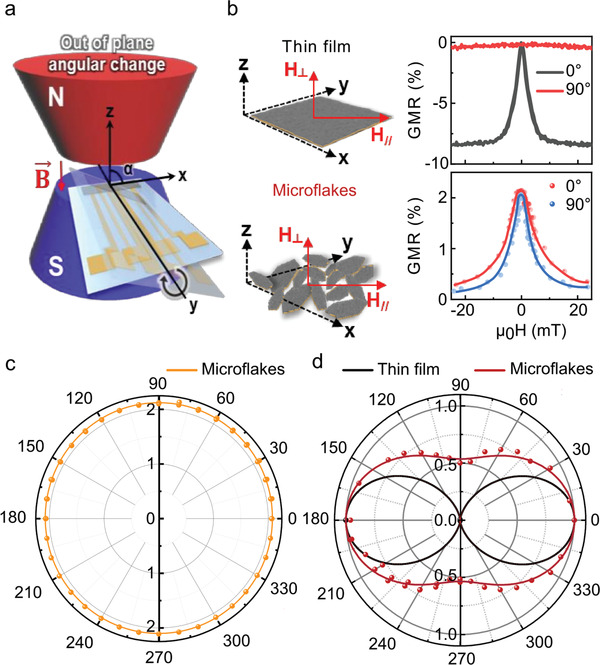
Angular‐dependent response of printed Py/Cu‐based GMR sensors. a) Schematics of the measurement setup revealing the rotation plane of the sensor with respect to the magnetic fields lines of an electromagnet. b) Comparison of the GMR performance between a thin film revealing GMR effect and printed GMR sensor with randomly oriented microflakes. The sensors are exposed to an in‐plane (0°) and out‐of‐plane (90°) magnetic field. c,d) Polar plots revealing the angular dependence of the GMR magnitude for a printed GMR sensor (orange curve) (c) and of the normalized sensor sensitivity for a thin film sensor (black curve) and printed GMR sensor (red curve) (d).

The application potential of highly compliant and printed GMR sensors for on‐skin electronics could be verified with the characterization of thermal stability. In particular, thermo‐mechanical behavior of binding polymers in printable pastes plays a significant role in determining operational temperature range of magnetic field sensors. Remarkable heat resistance of SBS triblock copolymer exhibits thermal decomposition over 400 °C.^[^ ^33^ ^]^ In addition, high glass transition temperature (*T*_g_ ≈100 °C) of polystyrene hard domains guarantees the applicability of our printed GMR sensors in broad temperature range from room temperature to 100 °C.^[^ ^37^ ^]^ Even though the higher thermal expansion coefficient of polymeric binders than inorganic substances is not preferable, loaded fillers in polymeric binders contribute to improve the thermal stability due to superior interference of heat dissipation from the distributed inorganic fillers.^[^ ^33^, ^47^, ^48^ ^]^ Therefore, our printed GMR sensors consisting of [Py/Cu]_30_ microflakes and triblock copolymer binder maintain magnetoresistive performance from 23 to 90 °C where the temperature range is enough to fulfil for operation of on‐skin and consumer electronics (**Figure** **4**a,b). Interestingly, when the temperature approaches to *T*_g_ of SBS, it causes 0.5% increase of GMR performance. We presume that the rubbery‐like behavior of thermoplastic SBS nearby *T*_g_ induces the reconfiguration of randomly oriented GMR microflakes to form a better percolation.

**Figure 4:**
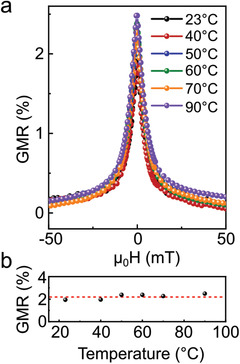
Temperature stability of printed Py/Cu‐based GMR sensors. a) GMR response after the printed sensor was exposed to successive heating cycles at different temperatures up to 90 °C. b) Variation of the GMR effect as a function of temperature.

These highly stable and printable GMR sensors can be readily applied for interactive electronics. We illustrate this concept with a demonstrator where the compliant GMR sensor was attached on a human fingertip and moved over a permanent magnet to control virtual objects on a computer screen (**Figure** **5**a). Under this configuration, the GMR sensor converts the finger position with respect to the magnet into voltage signals, which control the actions on‐screen. Two thresholds for action were defined; one that activates the zoom in/scroll up and one that triggers zoom out/scroll down function. Slightly approaching the sensor to the magnet produces a large enough voltage change to cross the pre‐defined threshold 1, resulting in a scroll up event. Further approaching to the magnet significantly increases the detected magnetic field and drives the sensor into threshold 2, which results in a scroll down event (Figure 5b,c). This kind of interaction was used to seamlessly navigate over a doc file (Figure 5c) and to zoom in and out of an interactive map pointing to the location of our host institute (Figure 5d). This demonstration highlights the potential of wearable and skin‐mountable magnetic sensors to enable remote control and touchless interactions in the low magnetic field range. We envision that these skin‐conformal and low‐field sensitive GMR sensors can broaden the applications of human‐machine interface systems without posing any risks to human health.

**Figure 5:**
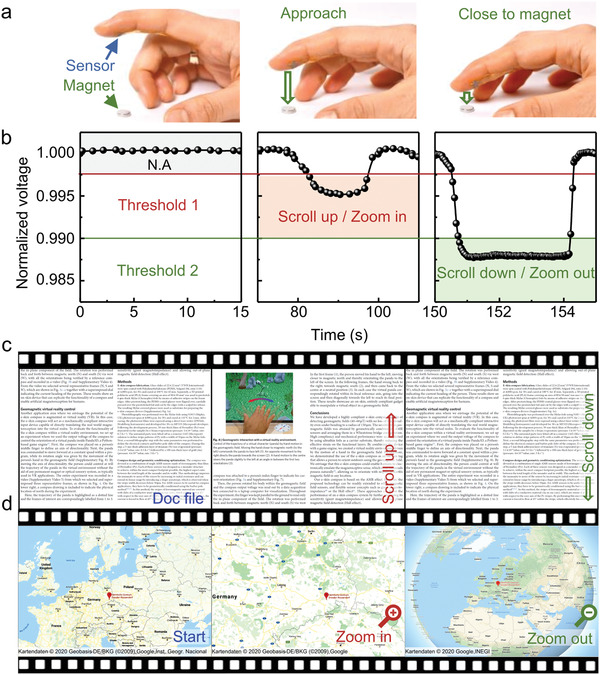
Highly compliant printed Py/Cu‐based GMR sensors for touchless interactive electronics. a) Photographs showing a compliant printed GMR sensor applied to a pointing finger. The finger is displaced with respect to a small permanent magnet, which results in the change of the sensor read out voltage. b) The time evolution of the normalized sensor read out dependent on the distance between the finger and the magnet. c,d) The sensor is connected to a PC, which displays either: c) a document (Movie S1, Supporting Information) or d) an interactive map (Movie S2, Supporting Information). The sensor signal is used to navigate through the document (scrolling) or zoom in/out the map. To realize interactivity, we define two threshold levels ((b), left image). By a moderate change of the sensor readout falling between the two thresholds ((b), middle image), the device allows scrolling up of a document ((c), transition from left to middle image) or zooming in into an interactive map ((d), transition from left to middle image) to be realized. When the signal change is larger than the second threshold ((b), right image), the device is programmed to scroll the document down ((c), transition from middle to right image) or zooming out an interactive map ((d), transition from middle to right image). The demonstrator with scrolling of the document is based on ref. ^[^ ^6^ ^]^: Reproduced with permission.^[^ ^6^ ^]^ Copyright 2018, The Author(s), published by Springer Nature Limited. For the demonstrator with the interactive control of a map, the images and video footage are taken from Google Maps with required attribution: Map data © 2019 Google, INEGI. Map data 2019 Geobasis‐DE/BKG (©2009).

In conclusion, we have demonstrated the very first printable and stretchable high performance magnetic field sensor relying on giant magnetoresistive (GMR) effect. Being printed on ultrathin polymeric foils, these sensor devices are capable of detection in low magnetic field of below 1 mT and sustaining high‐performance magneto‐resistive sensing under extreme mechanical deformation of up to 16 µm of bending radii and 100% stretching. The remarkable performance is achieved by dispersing GMR microflakes of [Py/Cu]_30_ in a viscous triblock copolymer based poly(styrene‐butadiene‐styrene) (SBS) elastomer. Owing to its supramolecular structure, SBS possesses high dissipative capacity and cohesive tear strength, resulting in strong adhesion of the magnetic composite to ultrathin polymeric foils. Benefiting from their unique mechanical compliancy, our printed GMR sensors can be easily applied on skin enabling wearable interactive electronics suitable for daily use by the public. We feature the potential of our highly‐compliant and printable magnetoresistive sensors in augmented reality settings, where a sensor‐functionalized finger conducts remote and touchless control of virtual objects manageable for scrolling electronic documents and zooming maps upon touchless interaction with a tiny permanent magnet.

Although the technology is demonstrated for GMR‐based devices, it can be readily extended to other magnetic as well as non‐magnetic functional elements. In particular, in the field of printable magnetoelectronics,^[^ ^49^, ^50^ ^]^ it is insightful to apply viscous triblock copolymers to realize printable and compliant high performance magnetic field sensors relying on anisotropic magnetoresistive effect^[^ ^6^ ^]^ and planar Hall effect^[^ ^51^ ^]^ revealing linear response to an external magnetic field. In this respect, the sensitivity of printed magnetic field sensors can be pushed even further down potentially reaching μT range and can be used for navigation purposes, as electronic switches for energy‐efficient interactive printed electronics for smart home applications.^[^ ^5^ ^]^ Furthermore, such configurations could spark prospective personal appliances that provide a ubiquitous interface between the physical world and augmented reality. To this end, these touchless interactive devices could open exciting possibilities for business, gaming, health‐monitoring, and fitness training.^[^ ^52^ ^]^ Taking advantage of the solution processability of the sensor devices, they can be manufactured in the same printing process with permanent magnets, which is attractive for the realization of smart magnetic soft robots with motion control based on the feedback from embedded magnetic field sensors. Ultimately, this technology can be extended toward more complex additive manufacturing approaches, where complex‐shaped mechanical structures^[^ ^53^, ^54^ ^]^ can be 3D printed with embedded high performance soft electronics.

## Experimental Section

### Preparation of [Py/Cu]_30_ and [Co/Cu]_50_ Microflakes

5 × 5 cm glass substrates were spin‐coated at 4000 rpm with AZ 1505 (MicroChemicals GmbH, Germany) and dried for 5 min at 90 °C. The resulting coating was used as a sacrificial layer in ensuing steps. Multilayered stacks of [Py(1.5 nm)/Cu(2.3 nm)]_30_ and [Co(1 nm)/Cu(2.2 nm)]_50_ coupled at the 2nd antiferromagnetic maximum were deposited on the coated substrates by magnetron sputtering at room temperature (Ar was used as a sputter gas; Ar pressure is 10^–3^ mbar; base pressure is 10^–7^ mbar; deposition rate is 2 Å s^−1^).

The sensitivity of a magnetoresistive sensor is primarily determined by two parameters: i) the actual magnetoresistive ratio and ii) the saturation field. By optimizing the material stack (thickness of layers, microstructure of layers, number of layers, choice of materials in the stack) it is possible to optimize both of these parameters to achieve the sensor performing in the magnetic field range of interest.^[^ ^55^ ^]^ In the case of Py/Cu multilayers coupled at the 2nd antiferromagnetic maximum, the optimization was performed in the direction of reducing the saturation field (bringing it as close to zero as possible), which was achieved on the expense of having somewhat reduced GMR. For the samples, GMR ratio is about 8%, which is smaller than that of Co/Cu multilayers coupled at the 1st antiferromagnetic maximum of about 55% used in prior studies.^[^ ^30^ ^]^ However, the saturation field of Co/Cu multilayers coupled at the 1st antiferromagnetic maximum is about 400 mT. It means, that these sensors are very well suited for measurements of magnetic fields in the range of 100 mT or so, which are relevant for industrial machines as an example (Figure 2). After printing, the saturation field of the Co/Cu‐based sensors coupled at the 1st antiferromagnetic maximum increases to more than 600 mT, ^[^ ^30^ ^]^ which does not let these sensors to operate in the field range relevant for on‐skin electronics. Accordingly to WHO, the field should be smaller than 40 mT. In contrast to Co/Cu stacks coupled at the 1st antiferromagnetic maximum, Py/Cu multilayers coupled at the 2nd antiferromagnetic maximum have saturation field of less than 20 mT, which allows to have much larger sensitivity in the low field region. In this respect, one of the key achievements of this work was that even after printing the sensor reveals its highest sensitivity at about 1 mT field was demonstrated, which is exactly the field range, which is relevant for on‐skin electronics to address interactive applications.

In addition to Py/Cu stacks coupled at the 2nd antiferromagnetic maximum, Co/Cu stacks coupled at the 2nd antiferromagnetic maximum (Figure 2) were prepared. It was seen that by using Co instead of Py and keeping all other processing steps the same, printed magnetic field sensors can be obtained with the sensitivity in the range of about 10 mT.

After deposition of GMR stacks, the ultrathin photoresist layer was removed in an ultrasonically excited acetone bath to lift‐off the metallic multilayers and transform them in microflakes (ultrasonication for 10 min). After cleaning the [Py/Cu]_30_ and [Co/Cu]_50_ microflakes with acetone and drying in an oven at 60 °C for 1 h, a powder of GMR microflakes was obtained.

Figure S6a (Supporting Information) compares the GMR response of single and chained flakes after lift‐off. An individual GMR flake maintains a comparable GMR response with respect to the thin‐film system (a slight decrease of about 7% with respect to the thin film value is due to a less defined geometry of the electrical contacts used for the measurement on the flake. On the other hand, a system of 3 mechanically contacted flakes (one flake indicated as “Flake 3” is just placed on top of two other flakes; Figure S6b,c, Supporting Information) shows a reduced GMR performance, amounting to 65% of the thin film value.

Additionally, the nominal 4‐point resistance of the system was increased from 0.9 Ω for a single flake to 3.7 Ω for the chained system. This experiment suggests that the reduction in the magnetoresistive ratio is due to the addition of the contact resistances at the interfaces between flakes. The electrical contact resistance contribution to the nominal resistance further increases for the paste system where the effective contact area between flakes is reduced, resulting in a lower GMR ratio of the printed sensors.

The experiment reported in Figure S6 (Supporting Information) demonstrates that the GMR ratio decreases due to additional contact resistance contribution at the interface between flakes rather than due to the degradation of the GMR performance of delaminated flakes.

### Magnetic Paste

To prepare magnetic paste, the as‐prepared GMR powder was mixed with a binding elastomer. As a binder, poly(styrene‐butadiene‐styrene) (SBS, Sigma‐Aldrich, Germany) was used, which is one of the thermoplastic elastomers showing rubber like physical property at room temperature. First, SBS was dissolved in Xylol with 0.15 g mL^−1^ of concentration at room temperature. After the SBS solution became optically transparent upon magnetic stirring for 12 h, it was mixed with the GMR powder at the concentration of 40 mg mL^−1^. This magnetic paste was applied using screen printing on a target substrate. Typical screens with a dimension of 3 × 2 mm^2^ were used in this study. The printed GMR sensors were ready after drying of the printed magnetic paste for at least 3 h at ambient. The highly compliant GMR sensors was printed on both ultrathin polyimide film (HD MicroSystems, USA) and Mylar foil (Chemplex Industries, USA).

### Wettability and Adhesion Tests

A Drop Shape Analyzer DSA25 (KRÜSS, Germany) was used to characterize the contact angle of binders with the GMR stack surface. The contact angle between the substrate and the droplets was measured using the tangential calculation module of the ADVANCE (KRÜSS, Germany) software. The acquisition mode of the DSA25 device was used to obtain cross‐sectional views of the adhesion behavior of a 5 mm × 30 mm PET probe attached to the GMR stack surface with SBS and PCH binders.

### Stretchable GMR Sensor

To develop the stretchable GMR sensor, the compliant and printed GMR sensor was mounted on the 100% of biaxially stretched VHB tape by using the home‐built stretching stage. After mounting GMR sensor, the Cu wires were connected with four‐electrode pad by Ag paste and dried for 1 h. The buckled structures were formed when the strain slowly lost from 100% to 0% strain rate. The resistance change of GMR sensor was verified with a B2902A tabletop multimeter (Keysight Technologies, USA) corresponding to the strain rate from 100% to 0%.

### Printed Interconnects

The thermal and magnetoresistive properties of the paste were measured using four‐point electrodes patterned on commercial printed circuit boards (PCBs) coated with photoresist (Bungard 120306E33‐10, Germany). The electrode patterns were cut from vinyl foil (Oracal 751, Orafol, Germany) sheets with an electronic cutter (Silhouette Portrait, USA) and transferred to poly(ethylene terephthalate) (PET; Mylar) foils to establish photolithographic masks. The PCBs were exposed through the masks with a UV illumination device (proMa 140 017, Germany), developed in a 10 g L^−1^ aqueous sodium hydroxide (VWR, Germany) solution and etched in a 2 g mL^−1^ iron chloride hexahydrate (Merck, Germany) solution. Following this procedure, the resulting boards were cleaned in acetone to remove any photoresist traces.

Flexible interconnects were fabricated by coating Ag nanowires (Sigma‐Aldrich, Germany) on ultrathin polyimide and Mylar foils. Electronic cut vinylvide foils with adhesive backing were employed as masks to transfer the four‐point electrode patterns to the ultrathin foils. After O_2_ plasma treating (40 W, 20 s) the exposed area of the ultrathin foils, a Ag nanowire suspension was coated by drop‐casting on the functionalized surface and dried at room temperature. Peeling off the vinyl foils completed the transfer process.

### Magnetoresistive Characterization

The magnetic response of compliant printed GMR sensors was characterized using an electromagnet by applying an in‐plane magnetic field, μ_0_ *H*_ext_, up to 200 mT. The coil was powered by a bipolar power supply (Kepco, USA). The longitudinal resistance of the printed sensors was measured in a 4‐wire configuration, using a Tensormeter (HZDR Innovation, Germany). Frequency and amplitude of the driving current were 775 Hz and 100 µA, respectively. The GMR ratio is defined as the magnetic field dependent change of the sample's resistance, *R*(*H*_ext_), normalized to the value of resistance when the sample is magnetically saturated, *R*_sat_: GMR(*H*_ext_) = [*R*(*H*_ext_) – *R*_sat_]/*R*_sat_. The sensitivity of the sensor element is defined as the first derivative of the sample's resistance over the magnetic field divided by the resistance value, *R*(*H*_ext_): *S*(*H*_ext_) = [d*R*(*H*_ext_)/d*H*_ext_]/*R*(*H*_ext_).

### Mechanical Characterization

The mechanical stability of the sensors was characterized upon static bending tests. The printed sensors were placed in between pole shoes of an electromagnet and mounted on different curved sample holders with curvature radii ranging from 16 µm to 5 mm. To ensure uniform field in the sensing plane, the sensors were mounted with their curvature axes perpendicular to the pole shoes axis. The magnetic field of the electromagnet was swept between −50 and 50 mT and the GMR response of the sensors was recorded. For the stretching test, the compliant GMR sensor was mounted on the 100% of biaxially stretched VHB tape. After mounting the GMR sensor, the Cu wires are connected with a four‐electrode pad using a commercial silver paste. The resistance change was verified with a multimeter (Keysight Technologies).

### Thermal Characterization

Printed GMR sensors were placed on top of a Peltier element (Tru components, Germany), which was attached with a thermal paste to an aluminum heat sink. A thermocouple was fixed to the top of the sensor to monitor the temperature during the heating experiment. By controlling the input voltage to the Peltier element, the samples were heated to the desired temperature (23–90 °C) and kept at constant temperature for 30 s before cooling down. Upon reaching room temperature, the GMR response of the sensor was measured. This procedure was repeated after every heating and cooling cycle to oversee any change in the GMR performance.

### Touchless Interactive Electronics

The signal processing algorithm shown in the Movies S1 and S2 (Supporting Information) consisted of two signal levels: LOW (marked as an upper, red horizontal line) and HIGH (lower, green horizontal line). In the absence of an external magnetic field (background signal), the program does not execute any command. Only if the signal reaches or exceeds the first threshold value (threshold LOW), the information displayed on the computer screen begins to scroll down for a displayed document (Movie S1, Supporting Information). If the finger continues to move further, the field will increase as the sensor goes closer to the permanent magnet and the signal level crosses the threshold defined as “HIGH”. This will cause the page to move in the opposite (scroll‐up) direction. In this demonstrator, the possible scenarios is limited by choosing 3 signal levels: “LOW,” “HIGH,” and “background” (N.A. = No Action). The last one is generated by Earth's magnetic field and the magnetic field of the surroundings.

A similar algorithm was applied to control the map zooming, shown in the Movie S2 (Supporting Information). In this case, a gentle movement of the finger decorated with a printed GMR sensor in the magnet direction reduced the scale (zoom‐in) while more pronounced motion with a significantly higher amplitude, increase the scale (zoom‐out).

The hardware for demonstrator was composed of several elements as described in Figure S7 (Supporting Information). The signal from the printed GMR sensor, attached to the pointing finger was generated while 1 mA current was passed through the magnetoresistor. Furthermore, the amplified analog signal was converted to digital with 24‐bit resolution and transmitted via USB interface to the computer by MyRIO (National Instruments, USA) card. The software was developed in LabVIEW environment where “HIGH,” “LOW,” and “Background” thresholds were defined. Depending on the strength of the signal, the program generated a command to control scrolling e‐documents or map zooming in real time.

### Use of On‐Skin Electronics

The measurements using on‐skin electronics were performed with the consent of all volunteers who participated in the study.

## Conflict of Interest

The authors declare no conflict of interest.

## Supporting information

Supporting Information

Supplemental Movie 1

Supplemental Movie 2
