# Supplementary material for: Printable and Stretchable Giant Magnetoresistive Sensors for Highly Compliant and Skin‐Conformal Electronics
Source: Adv Mater. 2021 Feb 2;33(12):2005521. doi: 10.1002/adma.202005521 (PMC11469064; doi:10.1002/adma.202005521)
Supplement: Supplementary file 1 — Supporting Information [file ADMA-33-2005521-s001.pdf]

© 2021 Wiley-VCH GmbH

ADVANCED MATERIALS

Supporting Information

for *Adv. Mater.*, DOI: 10.1002/adma.202005521

Printable and Stretchable Giant Magnetoresistive Sensors for Highly Compliant and Skin-Conformal Electronics

*Minjeong Ha, Gilbert Santiago Cañón Bermúdez, Tobias Kosub, Ingolf Mönch, Yevhen Zabila, Eduardo Sergio Oliveros Mata, Rico Illing, Yakun Wang, Jürgen Fassbender, and Denys Makarov**

Supporting Information

Printable and Stretchable Giant Magnetoresistive Sensors for Highly Compliant and Skin-Conformal Electronics

*Minjeong Ha, Gilbert Santiago Cañón Bermúdez, Tobias Kosub, Ingolf Mönch, Yevhen Zabila, Eduardo Sergio Oliveros Mata, Rico Illing, Yakun Wang, Jürgen Fassbender, Denys Makarov**

Supporting Movies:

Movie S1. Remote control for scrolling up and down of a document. Text and figure taken from a previously published work.[6] Reproduced with permission.[6] Copyright © 2018, The Author(s), under exclusive license to Springer Nature Limited.

Movie S2. Remote control for zoom in map (.wmv). Video footage taken from Google maps with required attribution as shown in the video. Map data © 2019 Google, INEGI. Map data © 2019 Geobasis-DE/BKG (©2009).

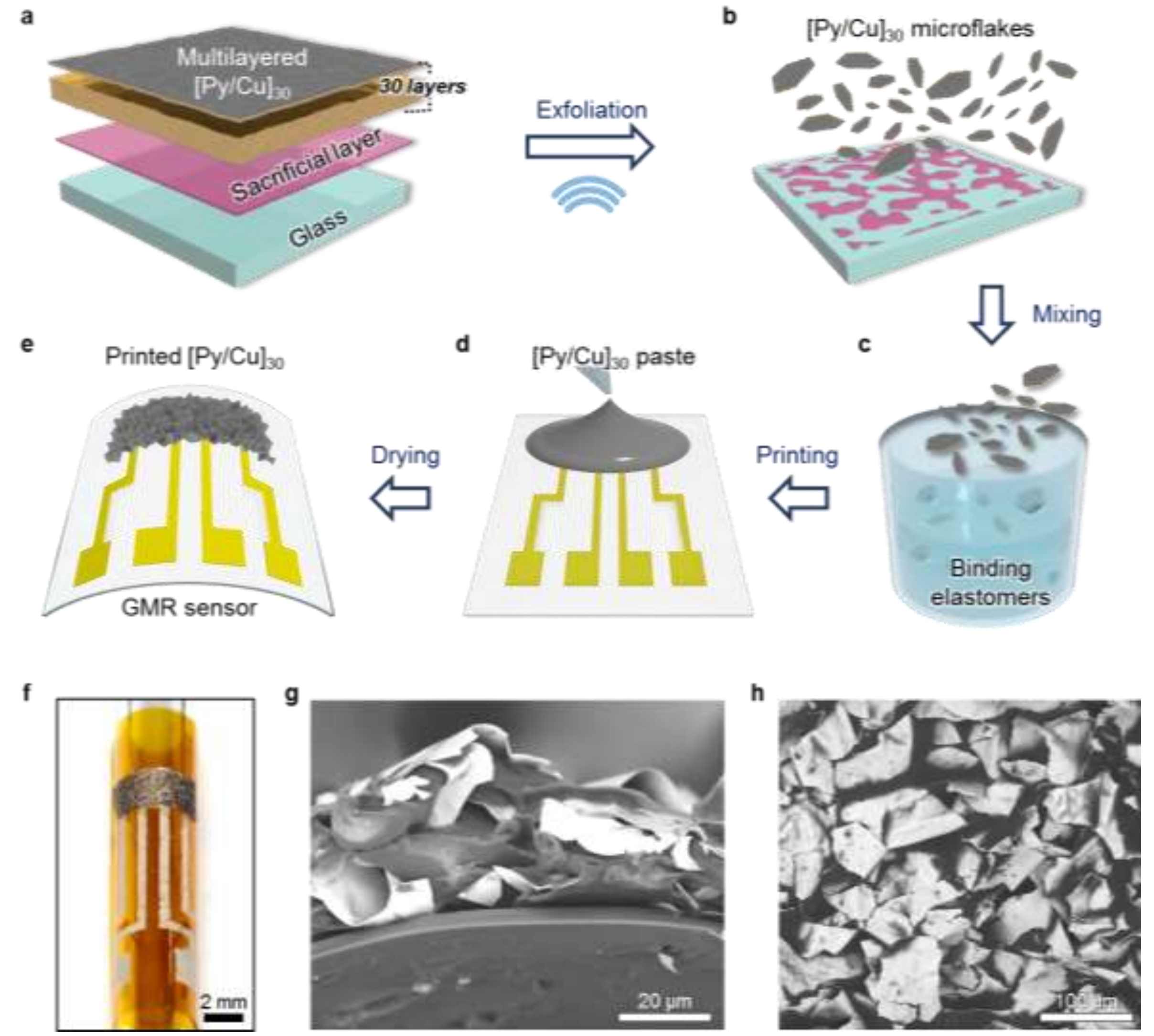

Figure S1. Fabrication of printed GMR sensors. (a) Deposition of $[Py/Cu]_{30}$ multilayers coupled at the 2nd antiferromagnetic maximum on top of the sacrificial photoresist coated glass substrates. (b) Exfoliation of GMR stacks by removing photoresist and transforming into microflakes under ultrasonically excited acetone. (c) Mixing GMR microflakes into binding elastomers (tribolock copolymer solution) for viscous magnetic inks. (d) Printing magnetic inks by screening square shape mask on top of the contact electrodes. (e) The highly compliant and printed GMR sensor could be acquired after drying it on 60 ^{o}C oven. (f) Curved GMR sensor on the cylinder with 1 mm of bending radius. (g) Cross-sectional SEM image of the tightly bonded GMR microflakes with SBS triblock copolymer. (h) Top view of SEM image of randomly distributed GMR microflakes of printed GMR sensor.

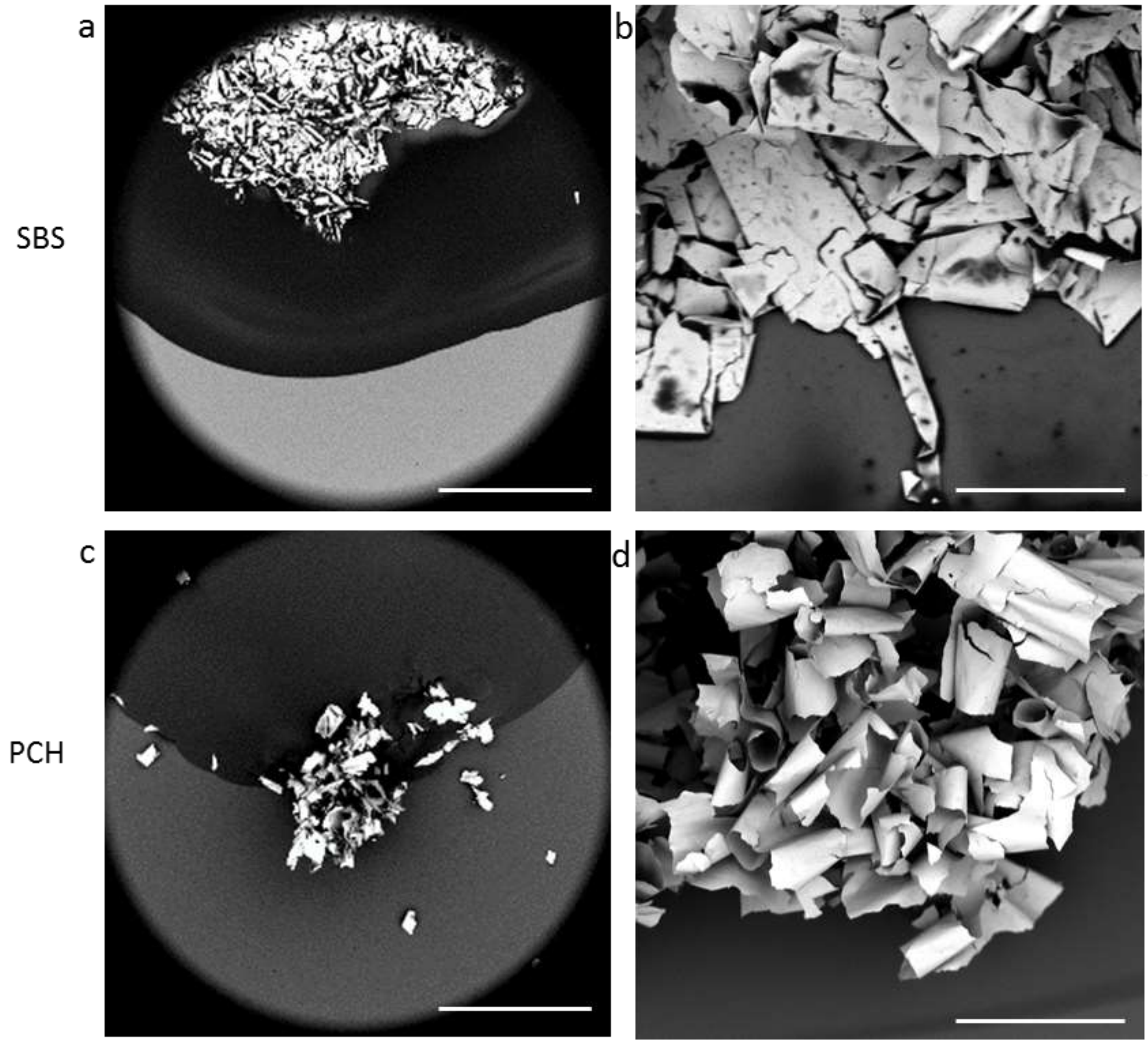

Figure S2. Flakes distribution on a binder droplet. (a) Flakes distribution after adding a 4 μL SBS droplet over sprinkled powder (Scale bar, 500 μm) and (b) after mechanically stirring the paste formulation (Scale bar, 100 μm). (c) Flakes distribution after adding a 4 μL PCH droplet over sprinkled GMR flakes (Scale bar, 500 μm) and (d) after mixing the paste formulation (Scale bars, 100 μm).

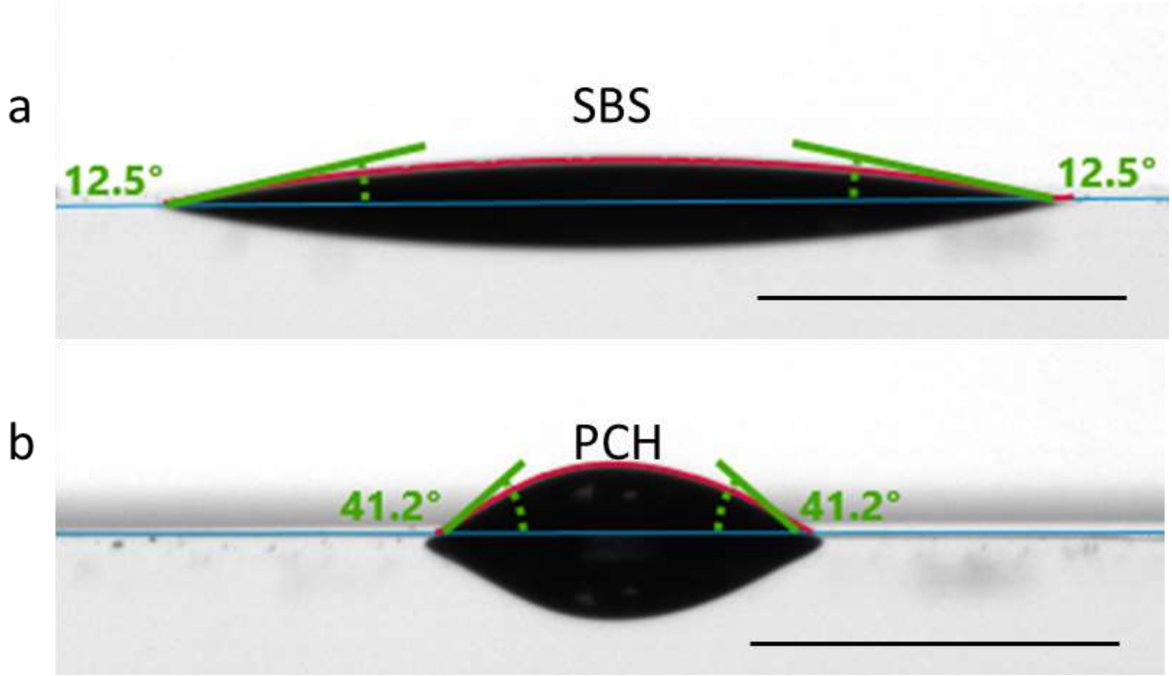

Figure S3. Wettability of binders with GMR stacks. Contact angle measurement of 7 μL droplets of (a) SBS and (b) PCH over a sample stack (SiOx wafer deposited with a $[Py/Cu]_{30}$ stack). Scale bar, 5 mm.

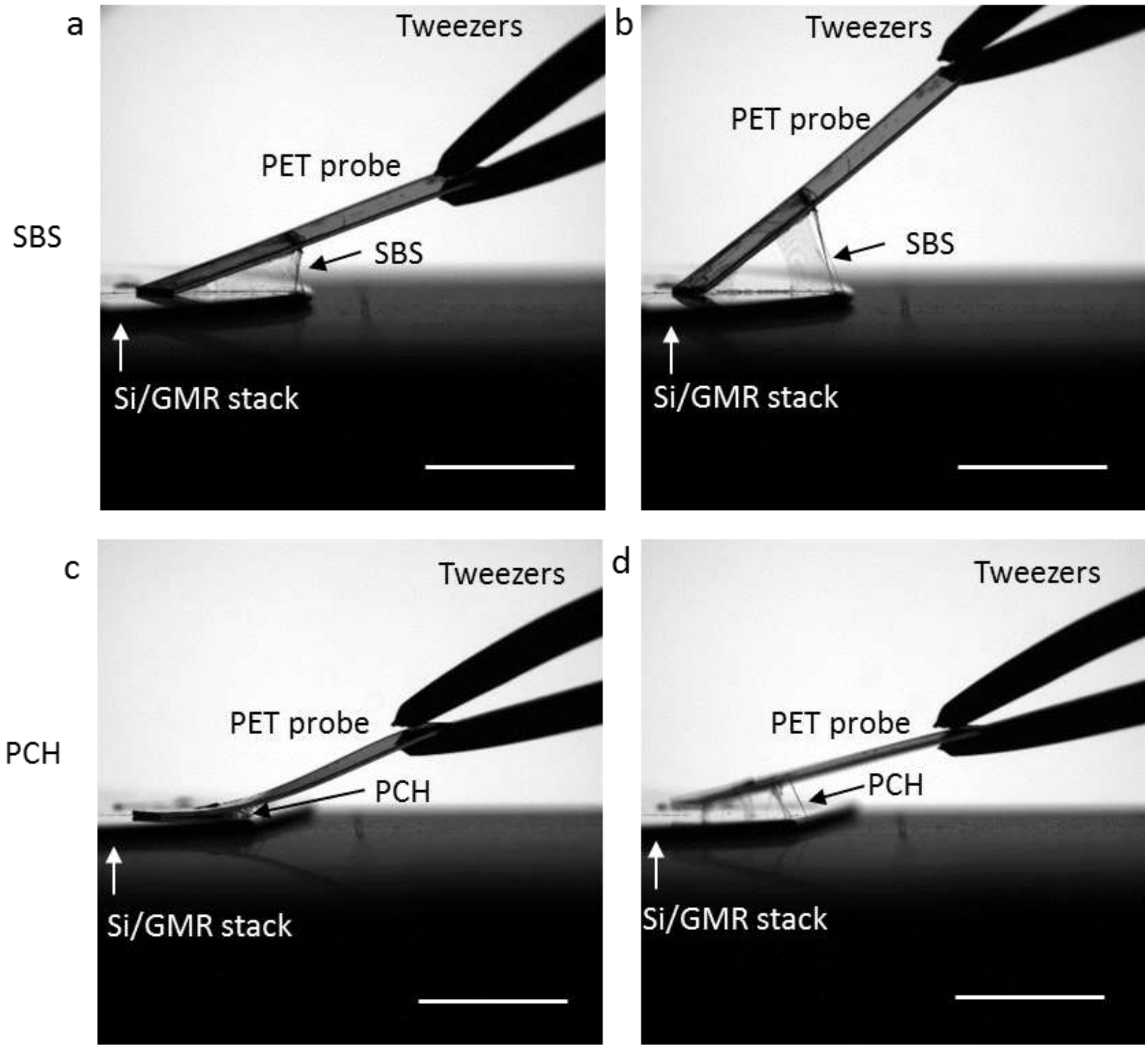

Figure S4. Peeling resistance test of binders. Peeling test showing (a) the force dilution of SBS and (b) high elasticity of the binder. (c) PCH shows reduced dissipation of the strain and (d) adhesive failure at lower peeling angle. Scale bars, 10 mm.

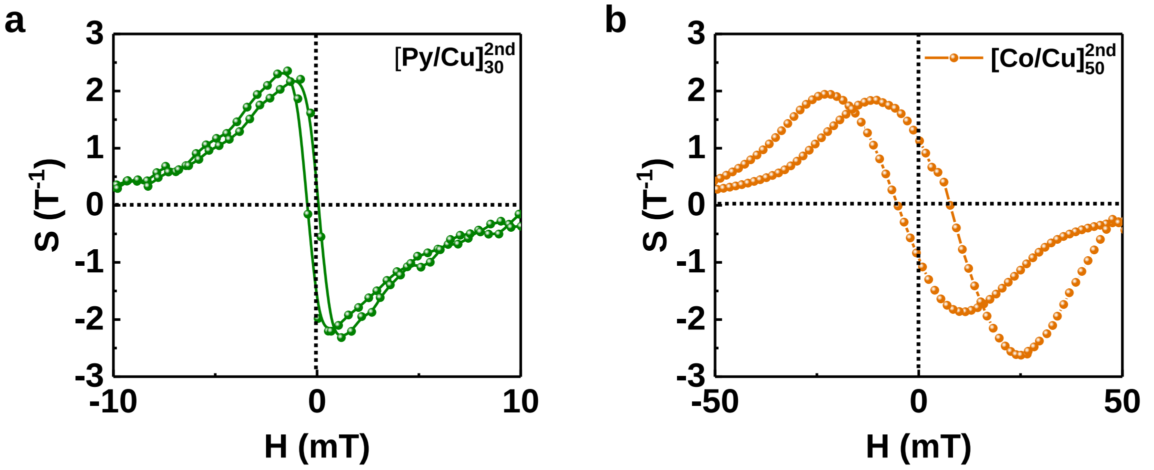

Figure S5. Variations of sensitivity depending on the magnetic field range (a) $[Py/Cu]_{30}$ and (b) $[Co/Cu]_{50}$ coupled at the 2nd antiferromagnetic maximum.

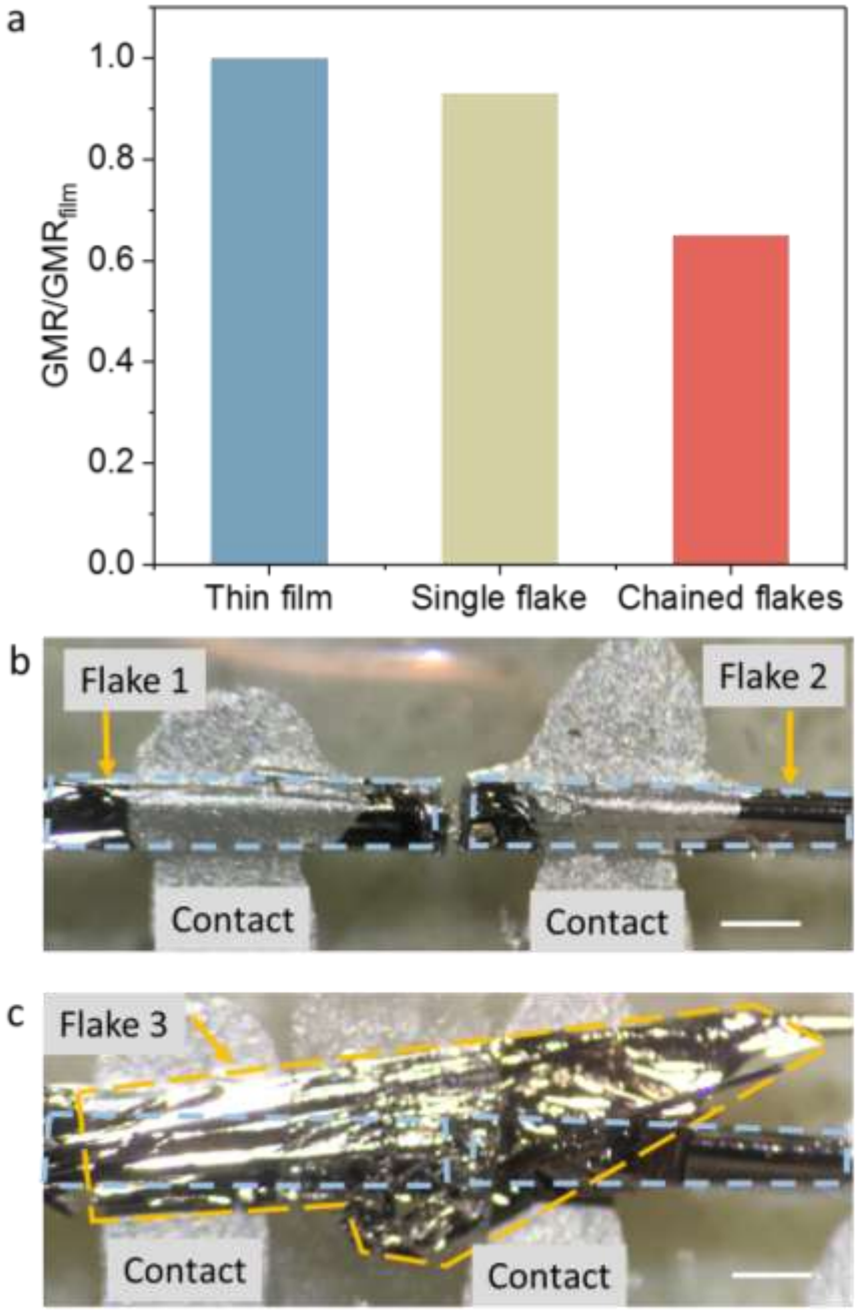

Figure S6. GMR performance of single and chained flakes. (a) GMR normalized with respect to the magnetoresistive response of thin films. After lift-off, a single flake maintains 93% of the GMR amplitude. The normalized GMR response of 3 chained flakes is reduced to 65% after (b) fixing each of the two GMR flakes ("Flake 1" and "Flake 2") with a silver paste contact and (c) connecting them through a third flake ("Flake 3"), which is mechanically attached to the two flakes. Scale bar, 2 mm.

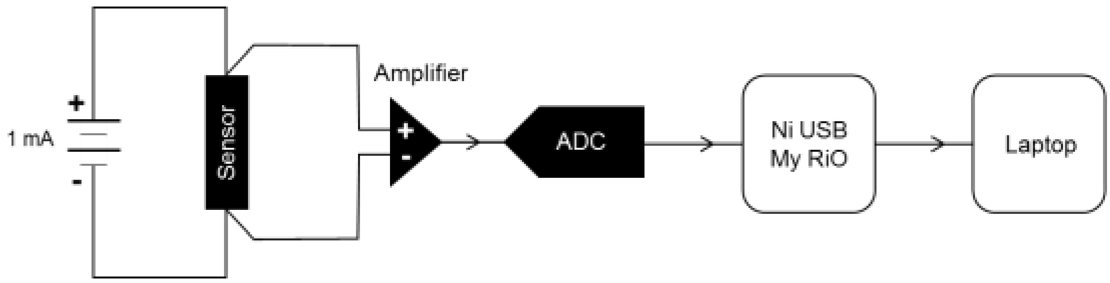

Figure S7. The hardware for demonstrator was composed of a GMR sensor, an analog-to-digital amplifier, and a signal analyzing MyRIO with LabView software.
